# Genomic of *Eucalyptus globulus *for pulp and biofuels

**DOI:** 10.1186/1753-6561-5-S7-P126

**Published:** 2011-09-13

**Authors:** Sofia Valenzuela, Renán García, Juan Elissetche, Jaime Rodríguez, Regis Mendonca, Claudio Balocchi

**Affiliations:** 1 Universidad de Concepción, Brazil; 2 Genómica Forestal, Brazil; 3 Bioforest S.A., Brazil

## 

*Eucalyptus *is a species of great interest for the pulp and paper industry. Worldwide there are 20 million hectares planted and within Chile a total of 700.000 ha, being the main species grown for short-fibre pulp *Eucalyptus globulus*, which is extensively used for pulping due to its fast growth, high pulp yield, and good fiber properties. Genetic programs of this specie have been oriented in improving commercial traits as volume, growth and form and recently traits as pulp ability have been included. Understanding the genomics of wood formation and identifying genes that are responsible for the traits of interest is a major challenge. An increase in 2% of pulp yield in trees can translate in a large economical gain, but most importantly there will be less pressure for land. There are two ways of reaching this goal, one through the use of genetic engineering and the second by genetic improvement, in both cases it is important to understand and identify the main genes involved in conferring the traits of interest. To identify these genes, different studies have been carried out, mainly by using EST libraries, finding a large number of sequences. Although there is large information regarding the enzymes and genes involved in the lignin pathway, little information is available regarding other metabolic pathways as cellulose and hemicellulose, as well as genes responsible for traits as density, cell wall, among others. In this study a detailed phenotypic characterization of 100 different genotypes was made, determining pulp content, lignin, cellulose, hemicellulose and Syringyl/Guaiacyl (S/G) ratio, among others. By employing NIR models a larger number of *E. globulus* clones were characterized for the same properties. From the 300 genotypes studied, “contrasting genotypes”, with high (good) and with low (bad) density and pulp yield were selected. It was found that clones with high density and pulp yield had high glucan content, lignin rich in S units, high β-O-4 linkages and low lignin, and xylan content. Out of these contrasting genotypes, one of each, low and high pulp yield and density, were further studied to find candidate genes involved in pulp ability. An EST library was made for each genotype, which was sequenced by the 454 platform, giving a total of 21,000 sequences, out of which 250 were differentially expressed. Due to the low amount of Eucalyptus sequences available, 28% of these sequences blasted to ESTs from wine grape, 22% to poplar and less than 5% to eucalypts. From these sequences, genes involved in lignin pathway, cellulose biosynthesis as well a transcription factors were identified. F5H was one of the main genes studied, which was characterized at a biochemical and expression level.

